# PancreaGuide for Computed Tomography Report Interpretation and Perioperative Consultation in Pancreatic Cancer: Development and Retrospective Evaluation of a Knowledge-Augmented Large Language Model Agent

**DOI:** 10.2196/101607

**Published:** 2026-11-09

**Authors:** Tianrui Kuang, Rui Wang, Shaomin Zhang, Yunwei Sun, Jin Zhou, Zhong Wu, Bing Peng

**Affiliations:** 1 Division of Pancreatic Surgery, Department of General Surgery and Laboratory of Pancreatic Cancer, West China Hospital of Sichuan University, No. 37, Guoxue Lane, Wuhou District, CHENGDU, China, 86 17786132270; 2 Center of Gerontology and Geriatrics, National Clinical Research Center for Geriatrics, West China Hospital of Sichuan University, Chengdu, China

**Keywords:** pancreatic cancer, resectability assessment, large language model, perioperative education, AI

## Abstract

**Background:**

Accurate interpretation of pancreatic cancer status and resectability-related information, together with effective perioperative communication, remains a major challenge in pancreatic cancer care. In routine practice, unstructured computed tomography (CT) reports and complex clinical information may limit the accessibility and consistency of clinical information delivery and increase the communication burden on clinicians. Large language models (LLMs) offer new opportunities for clinical information support; however, existing applications are often task-specific and rarely combine CT report interpretation with perioperative consultation within a unified patient-oriented framework.

**Objective:**

This study aimed to develop and retrospectively evaluate PancreaGuide, a knowledge-augmented LLM agent designed to support clinician-supervised CT report-based interpretation of pancreatic cancer resectability-related information and standardized perioperative consultation.

**Methods:**

We developed PancreaGuide, an agent-based framework integrating structured prompting, retrieval-augmented generation, and a domain-specific pancreatic surgery knowledge base to support clinical information interpretation and perioperative consultation. A retrospective cohort of 444 patients who underwent contrast-enhanced CT at West China Hospital between 2023 and 2025 was analyzed. Three LLMs, ChatGPT, DeepSeek, and Gemini, were evaluated in both base and PancreaGuide-integrated configurations. CT report-based interpretation of resectability-related information was evaluated using accuracy, sensitivity, specificity, positive predictive value, negative predictive value, *F*_1_-score, receiver operating characteristic curves (ROCs), and decision curve analysis (DCA) using a surgery-based proxy reference. Perioperative consultation quality was evaluated across 5 dimensions: accuracy, completeness, consistency, safety, and robustness.

**Results:**

Higher classification metric point estimates were observed after integration of the PancreaGuide framework across all 3 backbone models. In CT report-based interpretation of resectability-related information, Gemini+ achieved the highest accuracy of 87.4% (95% CI 0.840‐0.902) and a sensitivity of 95.9% (95% CI 0.927‐0.978). All models showed improved sensitivity and specificity estimates after PancreaGuide integration, with improved agreement between model-generated interpretations and observed surgical management status. ROC and DCA suggested improved discrimination and estimated net benefit using the surgery-based proxy reference. In perioperative consultation, PancreaGuide-integrated models received higher expert-rated scores than their corresponding base models across all 5 evaluation dimensions. Gemini+ achieved the highest overall score (mean 3.88, SD 0.38), followed by ChatGPT+ (mean 3.8, SD 10.36) and DeepSeek+ (mean 3.75, SD 0.35). The greatest improvements were observed in completeness and robustness, with consistent gains across clinical consultation domains.

**Conclusions:**

PancreaGuide provides a unified, knowledge-augmented framework for clinician-supervised CT report-based interpretation of resectability-related information and perioperative information support across multiple LLM backbones. The CT-based findings should be interpreted as agreement with observed surgical management status rather than validation against an independently adjudicated anatomical resectability. PancreaGuide may serve as an auxiliary information interpretation framework to improve the accessibility and consistency of clinical information delivery, while it should not replace formal radiological assessment, multidisciplinary evaluation, or clinician judgment.

## Introduction

Pancreatic cancer is one of the most lethal malignancies of the digestive system, and surgical resection remains the only potentially curative treatment [1,2]. However, accurate assessment and communication of resectability-related information remain challenging in pancreatic cancer care [3]. Pancreaticoduodenectomy is technically demanding, with complex anatomy and a high risk of perioperative complications, requiring accurate preoperative assessment and substantial surgical expertise. The increasing adoption of laparoscopic pancreaticoduodenectomy has placed additional emphasis on precise preoperative anatomical evaluation and operative planning [4].

In clinical practice, assessment of treatment options relies substantially on imaging evaluation, particularly contrast-enhanced computed tomography (CT), which plays a critical role in assessing tumor-vascular relationships, including involvement of the portal vein, superior mesenteric artery, and common hepatic artery [5,6]. These imaging features contribute to multidisciplinary assessment of anatomical resectability and treatment planning [7]. However, these CT reports are typically presented as unstructured medical text containing a large amount of technical terminology and fragmented descriptions, making it difficult for patients to accurately understand their meaning [8]. As a result, patients often struggle to fully grasp the severity of their condition and the basis for treatment decisions [9]. Furthermore, the perioperative journey encompasses a spectrum of concerns—from surgical risks to postoperative nutritional recovery—that require an integrated approach to bridge the information gap between complex clinical guidelines and patient understanding.

Recent advances in large language models (LLMs) have demonstrated strong capabilities in natural language understanding and medical reasoning, offering new opportunities for clinical information support and medical text interpretation [10-12]. However, most existing studies focus on single tasks, such as medical question answering or report summarization, and lack a unified framework that integrates CT report-based clinical information interpretation with perioperative patient education [13,14]. To address this gap, we developed PancreaGuide, a knowledge-augmented LLM agent framework for pancreatic cancer. The framework was designed for 2 clinician-supervised use cases: CT report-based interpretation of resectability-related information and standardized perioperative consultation support. In this retrospective study, we aimed to answer 2 specific research questions: first, whether PancreaGuide improves model agreement with observed surgical management status when interpreting unstructured CT reports; and second, whether PancreaGuide improves expert-rated quality of perioperative consultation responses compared with base LLMs. We further evaluated the framework across multiple LLM backbones to assess its robustness and model-agnostic applicability. Direct effects on patient understanding, shared decision-making, treatment adherence, and clinical outcomes were not assessed.

## Methods

### Overall Framework

We developed PancreaGuide, a knowledge-augmented LLM agent framework designed to support clinician-supervised interpretation of pancreatic cancer–related CT information and standardized perioperative consultation. The overall framework is illustrated in Figure 1. The framework was developed as an information-support system, rather than a replacement for radiological assessment, multidisciplinary evaluation, or clinician judgment.

**Figure 1.**
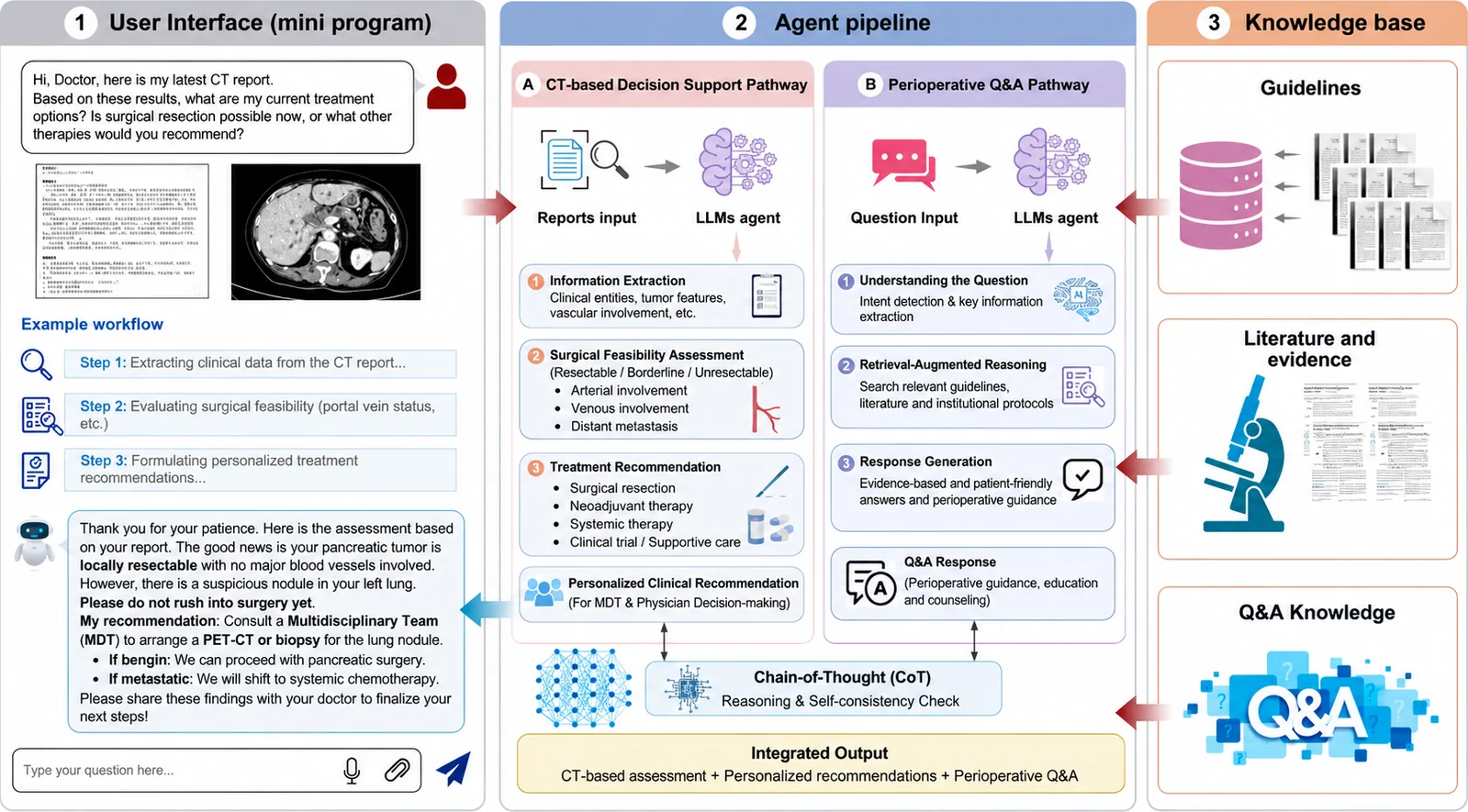
Architecture and workflow of the PancreaGuide clinical agent. The system integrates a user interface, an agent-based pipeline with computed tomography (CT) report–based interpretation and perioperative Q&A (question-answering) pathways, and a domain-specific knowledge base to generate structured clinical information outputs. CoT: chain-of-thought; LLM: large language model; MDT: multidisciplinary team; PET-CT: positron emission tomography-computed tomography.

PancreaGuide consists of two functional modules: (1) a CT report-based interpretation module that extracted and organized clinically relevant imaging information related to pancreatic cancer resectability, and (2) a perioperative question-answering (Q&A) module that generated patient-oriented clinical information based on a pancreatic surgery knowledge base.

Three general-purpose LLMs, including OpenAI GPT-5.4 Mini, Google Gemini-3-flash-preview, and DeepSeek-V3.2, were integrated as backbone models through Dify using the official API interfaces provided by the respective model developers. No parameter-level training or fine-tuning was performed. Instead, PancreaGuide enhanced the capabilities of these general-purpose LLMs through structured prompting, task-specific workflows, and knowledge-guided retrieval. Detailed model configurations, prompt templates, retrieval procedures, and postprocessing strategies are described in the Methods section in Multimedia Appendix 1, and model-specific technical information, including providers, model versions/IDs, and API access information, is summarized in Table S1 in Multimedia Appendix 1.

### Clinical Data Source and Patient Selection

We retrospectively identified consecutive patients who underwent contrast-enhanced upper abdominal CT examinations at West China Hospital, Sichuan University, between January 2023 and December 2025. A total of 519 patients were initially screened, including patients who underwent pancreatic surgery and those managed without surgery. Patients with pathologically confirmed nonpancreatic lesions after surgery were excluded.

After applying the predefined exclusion criteria, 444 patients were included in the final analysis. Observed surgical management status was used as a surgery-based proxy reference rather than a definitive anatomical gold standard for National Comprehensive Cancer Network (NCCN)–defined resectability. Patients who underwent surgical resection were categorized as the operated group, whereas patients managed without surgery were categorized as the nonoperated group. Because nonoperative management may be influenced by multiple factors beyond anatomical resectability, including patient preference, comorbidities, performance status, neoadjuvant therapy strategy, disease progression, institutional practice, and surgeons’ judgment, model performance was interpreted as agreement with observed surgical management status rather than direct validation of imaging-defined resectability. The corresponding preoperative CT radiology reports that are available before clinical management decisions were retrieved for analysis.

### CT Report Processing and Feature Extraction

All CT reports were obtained from routine clinical practice and were provided to PancreaGuide as unstructured Chinese text without manual restructuring [8]. The framework extracted clinically relevant imaging features, including tumor characteristics, vascular involvement, metastatic information, and local extension findings. Extracted vascular features included involvement of the portal vein, superior mesenteric vein, superior mesenteric artery, celiac axis, and common hepatic artery, together with supporting textual evidence from the original reports.

Based on the extracted information, PancreaGuide generated resectability-related interpretations according to NCCN-based categories, including resectable, borderline resectable, and unresectable disease. These outputs represented model-generated interpretations of written CT reports rather than independently adjudicated radiological classifications [15]. The overall workflow of CT-based feature extraction and resectability classification is illustrated in Figure 2.

**Figure 2.**
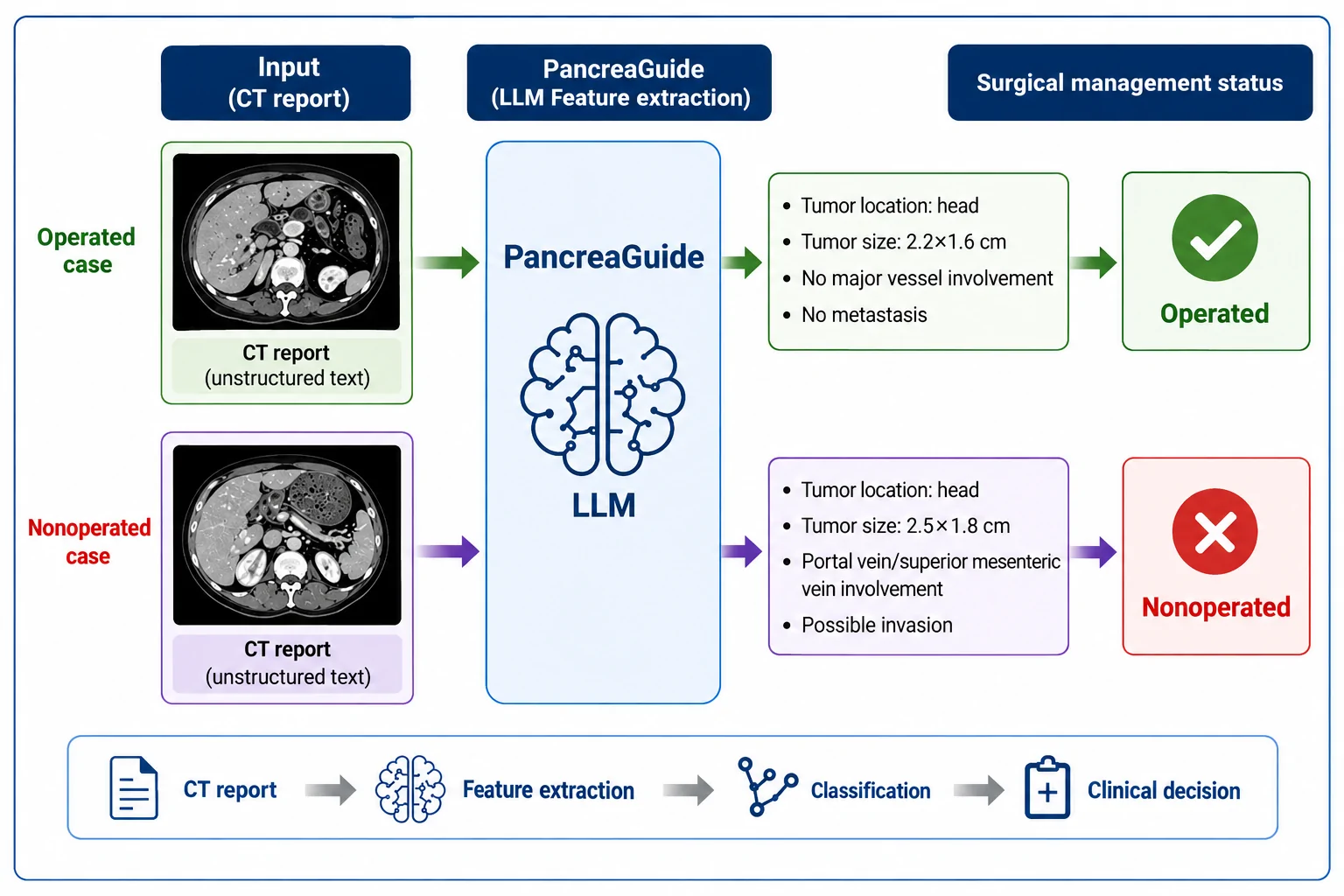
Workflow of computed tomography (CT) report–based feature extraction and resectability classification. Unstructured CT reports are used as input and processed by the PancreaGuide framework for structured feature extraction, including tumor location, size, vascular involvement, and metastatic status. Extracted features are subsequently mapped to predefined criteria to generate resectability-related interpretations (resectable, borderline resectable, or unresectable). Representative examples of different interpretation categories are shown. The overall pipeline illustrates the transformation from unstructured CT text to structured clinical information interpretation. LLM: large language model.

### Knowledge Base and Agent Workflow

A domain-specific pancreatic surgery knowledge base (PSKB) was constructed to support perioperative consultation [16-19]. The PSKB incorporated clinical guidelines, perioperative management protocols, and evidence-based literature covering pancreatic cancer diagnosis, treatment strategies, perioperative management, postoperative complications, nutrition, and follow-up [20-22].

A lightweight routing mechanism was used to identify whether incoming requests corresponded to CT report interpretation or to perioperative consultation. For CT-related tasks, the system performed structured information extraction and interpretation. For perioperative consultations, relevant knowledge segments were retrieved from the PSKB and incorporated into the generation context before response generation. The retrieval module used a hybrid strategy combining dense embedding similarity and sparse keyword matching.

A total of 62 standardized perioperative questions were developed to evaluate model-generated consultation responses across clinically relevant domains, including surgical management, postoperative recovery, complication management, diet and nutrition, and prognosis/follow-up (Table 1). These evaluation questions were developed independently from the knowledge-base Q&A pairs and were not directly included in the knowledge base using identical question-answer formulations. To assess the semantic retrieval capability of the retrieval-augmented generation (RAG) system, the evaluation questions were rephrased using clinically equivalent expressions, synonyms, and alternative descriptions rather than directly replicating the wording of the knowledge-base entries. The relevant medical knowledge remained available in the knowledge base to reflect realistic clinical application scenarios, where RAG systems are expected to retrieve and use domain-specific information. Detailed retrieval parameters, document preprocessing procedures, chunking strategies, context-injection procedures, and workflow specifications are described in the Methods section in Multimedia Appendix 1.

**Table 1.** Structured perioperative question set used to evaluate the PancreaGuide system.

ID	Category	Question
Q1	Surgery management	What is Laparoscopic Pancreaticoduodenectomy (LPD), and what are its core surgical steps and clinical indications?
Q2	Surgery management	For patients with borderline resectable pancreatic cancer receiving neoadjuvant chemo/radiotherapy, how is the optimal surgical window evaluated and determined?
Q6	Surgery management	Why do patients with severe obstructive jaundice typically require preoperative biliary drainage before LPD, and what is the pathophysiological rationale?
Q7	Surgery management	What is the target blood glucose range for diabetic patients prior to LPD? Why is perioperative glycemic stability critical for the prognosis?
Q8	Surgery management	How is the local infiltration and surgical resectability of pancreatic cancer evaluated clinically using comprehensive imaging and biochemical markers?
Q9	Surgery management	What is the clinical necessity of routine preoperative screening for Deep Vein Thrombosis (DVT) in the lower extremities before LPD?
Q49	Surgery management	What are the key points of perioperative management and high-risk assessment for octogenarian patients undergoing LPD?
Q4	Surgery management	What are the standard indications, such as intraoperative emergencies or anatomical limitations, that trigger a conversion to open surgery during LPD?
Q10	Surgery management	Why are multiple abdominal drainage tubes placed during LPD, and what specific postoperative complications does each tube monitor based on its anatomical location?
Q11	Surgery management	How is the benign or malignant nature of the tumor and surgical margins rapidly determined intraoperatively during LPD, such as through frozen section pathology?
Q12	Post-op recovery	How can intra-abdominal occult bleeding be identified early postoperatively through changes in vital signs, drainage characteristics, and laboratory metrics?
Q14	Post-op recovery	What does the multimodal analgesia protocol entail after LPD, and how should acute postoperative pain be effectively assessed and managed?
Q15	Post-op recovery	Why is it routine to measure the amylase concentration in the abdominal drainage fluid on postoperative days 1‐3, and what is its clinical significance?
Q16	Post-op recovery	What are the specific goals of early postoperative mobilization (under ERAS^a^ protocols), and how does it positively impact the prevention of cardiopulmonary and thrombotic complications?
Q21	Post-op recovery	How is the dynamic management of fluid input-output balance conducted within the first 24 hours postoperatively to prevent volume overload or tissue hypoperfusion?
Q22	Post-op recovery	What is the standard frequency for dressing changes of incisions and trocar sites after LPD, and what are the key indicators for observing wound healing?
Q52	Post-op recovery	What are the most critical vital signs and warning critical values that require close monitoring within the first 24 hours post-LPD in the intensive care unit?
Q3	Complication management	How is Postoperative Pancreatic Fistula (POPF) clinically graded (eg, Biochemical leak, grade B, and grade C) after LPD, and what are the standardized management principles for each grade?
Q5	Complication management	If a patient experiences sudden unexplained fever or severe abdominal pain after discharge, what late complications does this typically suggest, and what emergency steps should be taken?
Q13	Complication management	What is the pathophysiological mechanism of early postoperative hypoalbuminemia, what are its risks to tissue anastomotic healing, and how should it be appropriately corrected?
Q17	Complication management	How can a postoperative biliary leak be accurately identified through the color, volume, and bilirubin levels of the abdominal drainage fluid?
Q18	Complication management	What are the main causes of postoperative hypokalemia after LPD, and what are the clinical safety principles for intravenous and oral potassium supplementation?
Q19	Complication management	What is a postoperative chyle leak, and why does it typically manifest or worsen after the initiation of enteral nutrition or fat intake?
Q20	Complication management	What are the common causes of pleural effusion after LPD (eg, subphrenic inflammation and hypoalbuminemia), and what targeted interventions should be taken?
Q25	Complication management	What are the principles of step-up/step-down antibiotic therapy for preventing and treating perioperative infections? Why must the prolonged and blind use of broad-spectrum antibiotics be avoided?
Q26	Complication management	What local complication is typically indicated by erythema, pain, and exudate around the abdominal drain exit site, and what nursing interventions should be implemented?
Q28	Complication management	Besides Delayed Gastric Emptying (DGE), what other pathological factors (eg, paralytic ileus, intra-abdominal infection) must be ruled out for persistent postoperative abdominal distension?
Q29	Complication management	What is the standard emergency response protocol if an abdominal drainage tube is accidentally dislodged or displaced postoperatively?
Q30	Complication management	What is the typical laboratory threshold value for determining whether to administer intravenous human serum albumin to a postoperative patient?
Q40	Complication management	During the postoperative recovery period, how can adhesive intestinal obstruction be prevented through interventions? How should persistent distension and failure to pass gas or stool be managed in an emergency?
Q45	Complication management	What are the early warning signs and anticoagulant intervention strategies for portal vein or superior mesenteric vein thrombosis (PVT/SMVT) following LPD?
Q47	Complication management	What is the correlation between recurrent postoperative reflux cholangitis and bilioenteric anastomotic edema or stricture, and how is it clinically intervened?
Q48	Complication management	What is a marginal ulcer specific to post-LPD, what is its pathogenesis, and what is the prophylactic acid-suppression regimen?
Q50	Complication management	What is the prognosis for stress-induced Postoperative Atrial Fibrillation (POAF)? Do patients require long-term anticoagulant medications after discharge?
Q51	Complication management	What are the imaging characteristics and indications for percutaneous drainage of walled-off fluid collections near the surgical site or anastomosis post-LPD?
Q55	Complication management	What is the emergency protocol and criteria for interventional/surgical management in the event of sudden postpancreatectomy hemorrhage (PPH), presenting as massive hematemesis or melena?
Q56	Complication management	What are the common predisposing factors (eg, infection, electrolyte imbalance, and medications) and symptomatic treatments for postoperative delirium (confusion and hallucinations)?
Q57	Complication management	How can postoperative nausea and vomiting (PONV) be effectively prevented and managed in the perioperative period through pharmacological and nursing interventions?
Q58	Complication management	How should endocrine management be conducted for secondary diabetes (pancreatogenic diabetes) caused by partial resection of pancreatic tissue following LPD?
Q60	Complication management	What are the diagnostic criteria, early identification markers, and conservative medical management protocols for post-pancreatectomy acute pancreatitis (PPAP)?
Q23	Diet and nutrition	How is enteral nutrition scientifically maintained during delayed gastric emptying (DGE), and why is the retention of a nasojejunal feeding tube crucial for the patient?
Q24	Diet and nutrition	How is the improvement of DGE determined clinically? What are the reliable physiological signs for removing the nasogastric tube and transitioning to oral intake?
Q27	Diet and nutrition	What is pancreatic enzyme replacement therapy (PERT)? Why do patients who have undergone LPD typically require lifelong regular administration of pancreatic enzyme preparations?
Q31	Diet and nutrition	How can steatorrhea (frequent bowel movements and greasy stools) caused by postoperative exocrine pancreatic insufficiency be managed through dietary adjustments and PERT dose optimization?
Q39	Diet and nutrition	During home rehabilitation, what step-up dietary management strategy should the patient follow to prevent long-term weight loss and malnutrition?
Q44	Diet and nutrition	How should early screening and clinical intervention be conducted for secondary osteoporosis or vitamin deficiencies caused by malabsorption after LPD?
Q46	Diet and nutrition	What is the clinical significance of testing triglyceride levels in drainage fluid for diagnosing a chyle leak? Once diagnosed, how should a strict low-fat/fat-free medium-chain triglyceride (MCT) step-up diet be implemented?
Q54	Diet and nutrition	How is delayed gastric emptying (DGE) defined and graded clinically post-LPD? During DGE, what are the physiological advantages and nutritional maintenance protocols of jejunal tube feeding?
Q32	Prognosis and follow-up	What does “R0 resection” in a postoperative pathology report signify? Why is achieving microscopic negative margins critical to the overall survival prognosis of pancreatic cancer?
Q33	Prognosis and follow-up	How should the lymph node metastasis positivity rate in a pathology report be interpreted? How should targeted adjuvant therapy strategies be adjusted for patients with lymph node metastasis?
Q34	Prognosis and follow-up	What is the perineural invasion (PNI) characteristic of pancreatic cancer? Why is PNI positivity closely associated with refractory postoperative back pain and a high risk of local recurrence?
Q35	Prognosis and follow-up	What is the fundamental difference in biological behavior, invasiveness, and prognosis between “well-differentiated” and “poorly differentiated” tumors in histological grading?
Q36	Prognosis and follow-up	What is the optimal golden time window to initiate adjuvant chemotherapy following LPD? What are the negative impacts of delaying chemotherapy?
Q37	Prognosis and follow-up	Why does the dynamic trend (kinetics) of the tumor marker CA19-9 hold greater prognostic predictive value than a single absolute measurement during postoperative follow-up?
Q38	Prognosis and follow-up	What is the guiding role of tumor tissue genetic testing (eg, breast cancer susceptibility gene mutation profiling) in selecting personalized targeted or immunotherapy for pancreatic cancer postsurgery?
Q41	Prognosis and follow-up	Is persistent dull lower back pain occurring months or years postsurgery always a sign of local tumor recurrence or celiac plexus involvement? What diagnostic workup is required?
Q42	Prognosis and follow-up	During routine postoperative follow-ups, which imaging modalities (eg, contrast-enhanced MRI^b^) and biochemical markers are most sensitive in detecting early hepatic metastases?
Q43	Prognosis and follow-up	In long-term lifestyle management after LPD, why is absolute smoking cessation and alcohol restriction mandatory? What direct impact does this have on preventing recurrence and preserving remnant pancreatic function?
Q53	Prognosis and follow-up	If local regional recurrence is definitively detected during LPD follow-up, is there still an opportunity for secondary surgical exploration? What are the current mainstream non-surgical salvage treatment options?
Q59	Prognosis and follow-up	During home recovery, what are the “red flag” symptoms (eg, recurrence of jaundice, changes in drainage characteristics, and persistent severe vomiting) that patients’ families must recognize to warrant an immediate return to the emergency department?
Q61	Prognosis and follow-up	If CA19-9 levels show continuous progressive elevation during follow-up, but imaging such as PET/CT^c^ reveals no definitive recurrent lesions, how should the clinical strategy for further monitoring or intervention be formulated?
Q62	Prognosis and follow-up	For long-term survivors achieving a disease-free survival (DFS) of over 5 years post-LPD, besides routine tumor monitoring, what are the special areas of focus during follow-up (eg, incident fatty liver disease, and primary cancers)?

a ERAS: enhanced recovery after surgery.

b MRI: magnetic resonance imaging.

c PET/CT: positron emission tomography-computed tomography.

### Model Evaluation and Comparison Strategy

Model performance was evaluated separately for the CT report-based interpretation module and the perioperative Q&A module.

For CT-based interpretation, model outputs were compared with the observed surgical management status and categorized as true positive (TP), true negative (TN), false positive (FP), and false negative (FN). Accuracy, sensitivity, specificity, positive predictive value (PPV), negative predictive value (NPV), *F*_1_-score, receiver operating characteristic (ROC) curves, area under the curve (AUC), and decision curve analysis (DCA) were calculated.

For perioperative consultation, model responses were evaluated across 5 dimensions: accuracy, completeness, consistency, safety, and robustness. Each response was independently assessed by 3 senior pancreatic surgeons blinded to model identity and configuration. Interrater reliability for perioperative consultation scoring was assessed using a 2-way random-effects, absolute-agreement intraclass correlation coefficient (ICC) based on the mean ratings of 3 experts [ICC(2,3)], with 95% CIs. Both base models and PancreaGuide-integrated models (“+”) were evaluated under identical conditions using the same datasets and standardized inputs.

### Error-Source Analysis

A structured model–case error-source analysis was performed for FP and FN classifications generated by PancreaGuide-integrated models. Because the same patient could be misclassified by more than one model, the unit of analysis was defined as the model–case error event.

Each event was independently reviewed by 2 senior pancreatic surgeons based on the original CT report, structured vascular and metastatic descriptors, preoperative treatment information, model-generated classification, and observed surgical management status. Each event was assigned one primary source of disagreement, chosen from the following: ambiguous vascular involvement, incomplete or indeterminate metastatic reporting, borderline terminology or dichotomization, treatment-related changes after neoadjuvant therapy, discordance introduced by the surgery-based proxy reference, model extraction or interpretation error, or other/uncertain causes. Disagreements between reviewers were adjudicated by a third senior reviewer. Detailed adjudication procedures and error-source distributions are provided in the Methods section and Table S2 in Multimedia Appendix 1.

### Statistical Analysis

Statistical analyses were performed using R (R Foundation for Statistical Computing) and Python [9]. Continuous variables were compared using the Student *t* test or Mann-Whitney *U* test, as appropriate. Categorical variables were analyzed using the chi-square test. Performance metrics were reported with 95% CIs, where applicable. Interrater reliability for perioperative consultation scoring was assessed using a 2-way random-effects, absolute-agreement ICC based on the mean ratings of 3 experts [ICC(2,3)], with 95% CIs. Categorical agreement for error-source classification was evaluated using Cohen κ. A 2-sided *P* value <.05 was considered statistically significant.

### Ethical Considerations

This study was approved by the Ethics Committee of West China Hospital, Sichuan University (Approval Number 20250931) and conducted in accordance with the Declaration of Helsinki. The requirement for informed consent was waived due to the retrospective nature of the study. The study was prospectively registered in the Chinese Clinical Trial Registry (ChiCTR2600119597).

## Results

### Patient Characteristics

The patient selection process is illustrated in Figure 3. A total of 519 patients who underwent contrast-enhanced upper-abdominal CT were initially screened, and 444 patients were included in the final analysis after applying the predefined inclusion and exclusion criteria. Among the included patients, 245 (55.2%) underwent surgical resection, while 199 (44.8%) were managed nonoperatively. These groups were defined based on observed surgical management status and were used as a surgery-based proxy reference rather than as definitive NCCN-defined resectable and unresectable groups. The median age of the cohort was 61.5 (IQR 55.0‐69.0) years, and 59.5% (n=264) of patients were male. Tumors were predominantly located in the pancreatic head (n=270, 60.8%), followed by the pancreatic neck/body (n=122, 27.5%) and uncinate process (n=52, 11.7%). The median tumor size was 2.8 (IQR 1.8‐3.5) cm. Preoperative therapy was administered to 24.8% (n=110) of patients. Baseline characteristics of the study population are summarized in Table 2.

**Figure 3.**
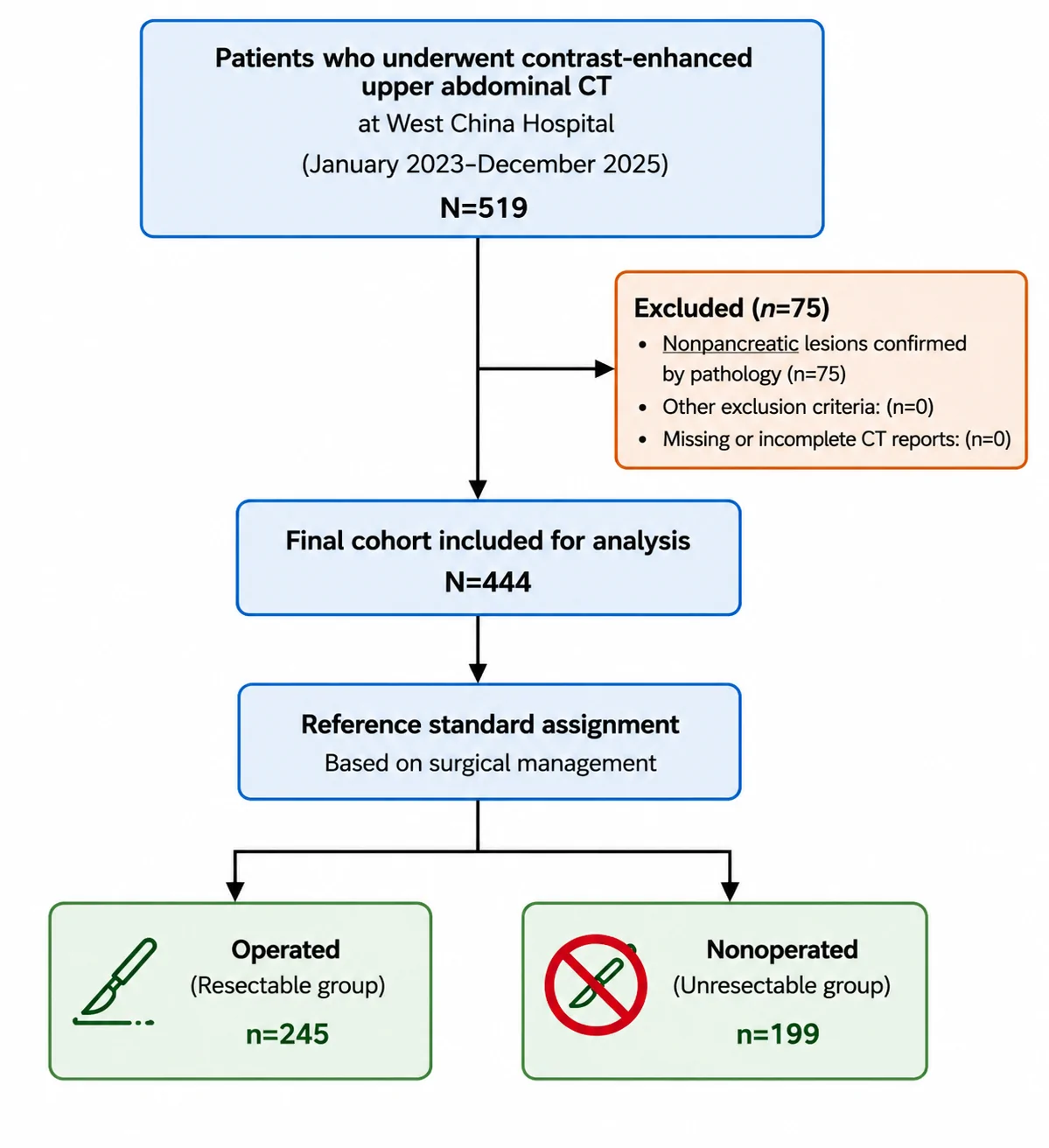
Flow diagram of patient selection and cohort construction. A total of 519 patients who underwent contrast-enhanced upper abdominal computed tomography (CT) were screened. After exclusion of 75 patients with nonpancreatic lesions, 444 patients were included in the final analysis. Observed surgical management status was used as a surgery-based proxy reference, with 245 patients classified as the operated group and 199 patients classified as the nonoperated group.

**Table 2.** Baseline characteristics of the study cohort.

Variable	Overall (n=444)	Operated (n=245)	Nonoperated (n=199)
Age (y), median (IQR)	61.5 (55.0‐69.0)	63.0 (54.0‐70.0)	61.0 (55.0‐67.5)
Sex, n (%)			
Male	264 (59.5)	136 (55.5)	128 (64.3)
Female	180 (40.5)	109 (44.5)	71 (35.7)
Tumor size (cm), median (IQR)	2.8 (1.8‐3.5)	2.7 (1.8‐3.2)	3.0 (1.8‐4.5)
Tumor location, n (%)			
Pancreatic head	270 (60.8)	163 (66.5)	107 (53.8)
Pancreatic neck/body	122 (27.5)	55 (22.4)	67 (33.7)
Uncinate process	52 (11.7)	27 (11.0)	25 (12.6)
Preoperative therapy, n (%)			
Yes	110 (24.8)	45 (18.4)	65 (32.7)
No	334 (75.2)	200 (81.6)	134 (67.3)

### Overall Diagnostic Classification Performance

The classification performance of different LLM configurations under the surgery-based proxy reference is summarized in Figure 4 and Table 3. As shown in Figure 4A, PancreaGuide models showed higher accuracy than their corresponding base models. Among base models, ChatGPT and Gemini demonstrated comparable accuracy (355/444, 80.0% and 359/444, 80.9%, respectively), whereas DeepSeek showed lower performance (300/444, 67.6%). After integration, accuracy increased to 85.8% (381/444) for ChatGPT+, 74.3% (330/444) for DeepSeek+, and 87.4% (388/444) for Gemini+.

**Figure 4.**
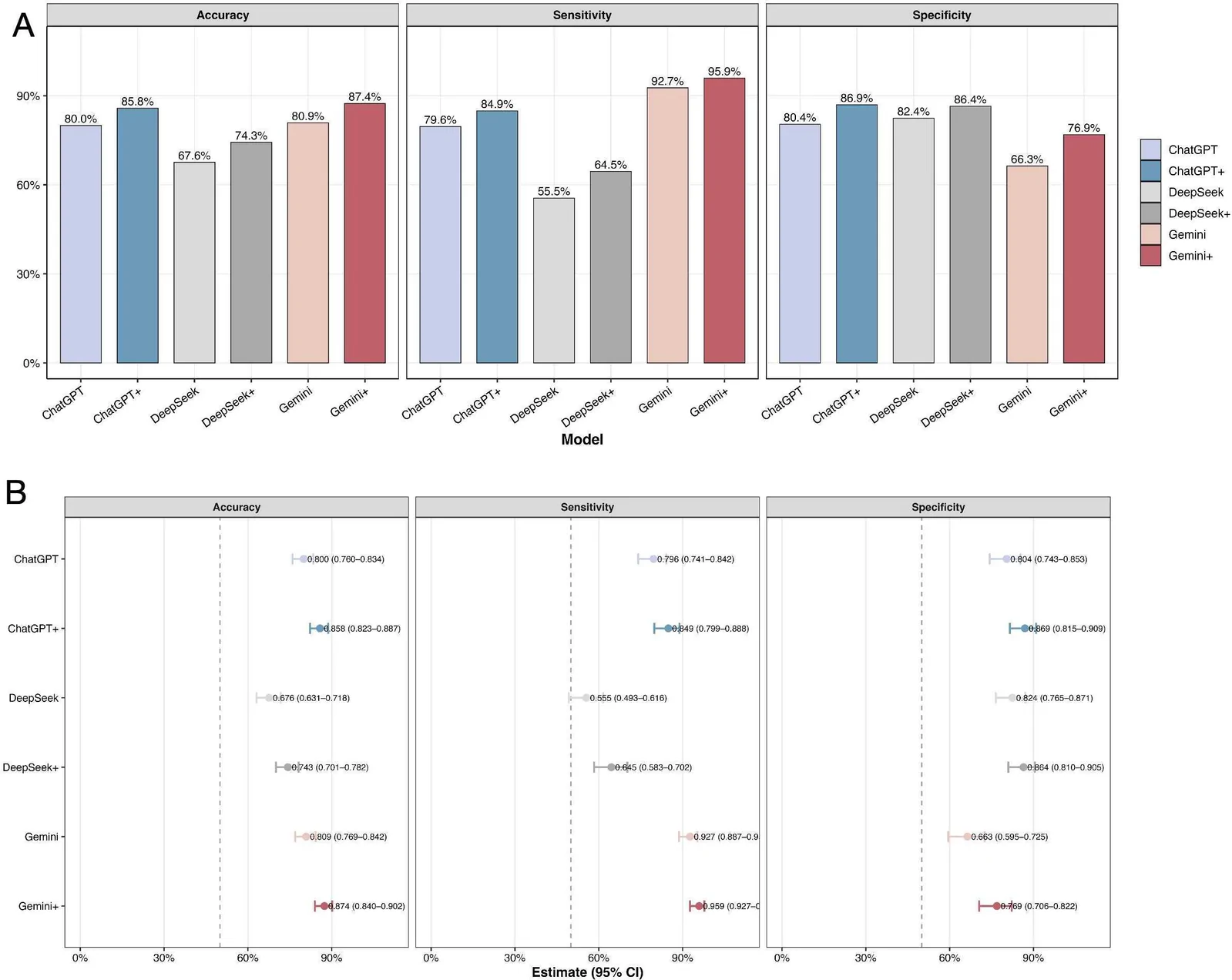
Performance of large language models in computed tomography (CT) report-based resectability-related classification. (A) Bar plots showing accuracy, sensitivity, and specificity for base models (ChatGPT, DeepSeek, and Gemini) and their corresponding PancreaGuide-integrated versions (denoted by “+”). (B) Forest plots showing point estimates and 95% CIs for each performance metric across models.

**Table 3.** Diagnostic performance of large language models in computed tomography (CT)–based resectability classification.

model_label	PPV^a^	NPV^b^	*F*_1_-score	Accuracy, (95% CI)	Sensitivity, (95% CI)	Specificity, (95% CI)
ChatGPT	0.833	0.761	0.814	0.800 (0.760‐0.834)	0.796 (0.741‐0.842)	0.804 (0.743‐0.853)
ChatGPT+	0.888	0.823	0.868	0.858 (0.823‐0.887)	0.849 (0.799‐0.888)	0.869 (0.815‐0.909)
DeepSeek	0.795	0.601	0.654	0.676 (0.631‐0.718)	0.555 (0.493‐0.616)	0.824 (0.765‐0.871)
DeepSeek+	0.854	0.664	0.735	0.743 (0.701‐0.782)	0.645 (0.583‐0.702)	0.864 (0.810‐0.905)
Gemini	0.772	0.880	0.842	0.809 (0.769‐0.842)	0.927 (0.887‐0.953)	0.663 (0.595‐0.725)
Gemini+	0.836	0.939	0.894	0.874 (0.840‐0.902)	0.959 (0.927‐0.978)	0.769 (0.706‐0.822)

a PPV: positive predictive value.

b NPV: negative predictive value.

Sensitivity improved after PancreaGuide integration across all models (Figure 4A). Gemini showed the highest baseline sensitivity at 92.7% (227/245; 95% CI 88.7%-95.3%), which further increased to 95.9% (235/245; 95% CI 92.7%-97.8%) after integration. ChatGPT showed an increase in sensitivity from 79.6% (195/245; 95% CI 74.1%-84.2%) to 84.9% (208/245; 95% CI 79.9%-88.8%), while DeepSeek increased from 55.5% (136/245; 95% CI 49.3%-61.6%) to 64.5% (158/245; 95% CI 58.3%-70.2%). Specificity also increased following integration, from 80.4% (160/199; 95% CI 74.3%-85.3%) to 86.9% (173/199; 95% CI 81.5%-90.9%) for ChatGPT, from 82.4% (164/199; 95% CI 76.5%-87.1%) to 86.4% (172/199; 95% CI 81.0%-90.5%) for DeepSeek, and from 66.3% (132/199; 95% CI 59.5%-72.5%) to 76.9% (153/199; 95% CI 70.6%-82.2%) for Gemini. PancreaGuide-integrated models exhibited higher point estimates across the evaluated metrics, as shown in Figure 4B. The corresponding 95% CIs generally overlapped.

### Prediction Consistency and Potential Informational Utility

The agreement between model predictions and observed surgical management status is illustrated using Sankey diagrams in Figure 5. As shown in Figure 5, PancreaGuide-integrated models demonstrated greater agreement with observed surgical management status compared with base models. In particular, Gemini+ showed a high proportion of positive classifications among patients who ultimately underwent surgery and showed a relatively low number of FN classifications under the surgery-based proxy reference. In addition, a substantial proportion of patients classified as borderline resectable were ultimately managed surgically, as illustrated by the transition flows in Figure 5. Confusion matrices are presented in Supplementary Figure 1 in Multimedia Appendix 1. As shown in Supplementary Figure 1C in Multimedia Appendix 1, Gemini+ demonstrated the lowest number of FN cases. Similarly, Supplementary Figure 1A in Multimedia Appendix 1 shows that ChatGPT+ showed higher specificity, with fewer FP classifications in the nonoperated cohort. Overall, PancreaGuide integration reduced both FN and FP predictions across all models under the surgery-based proxy reference.

**Figure 5.**
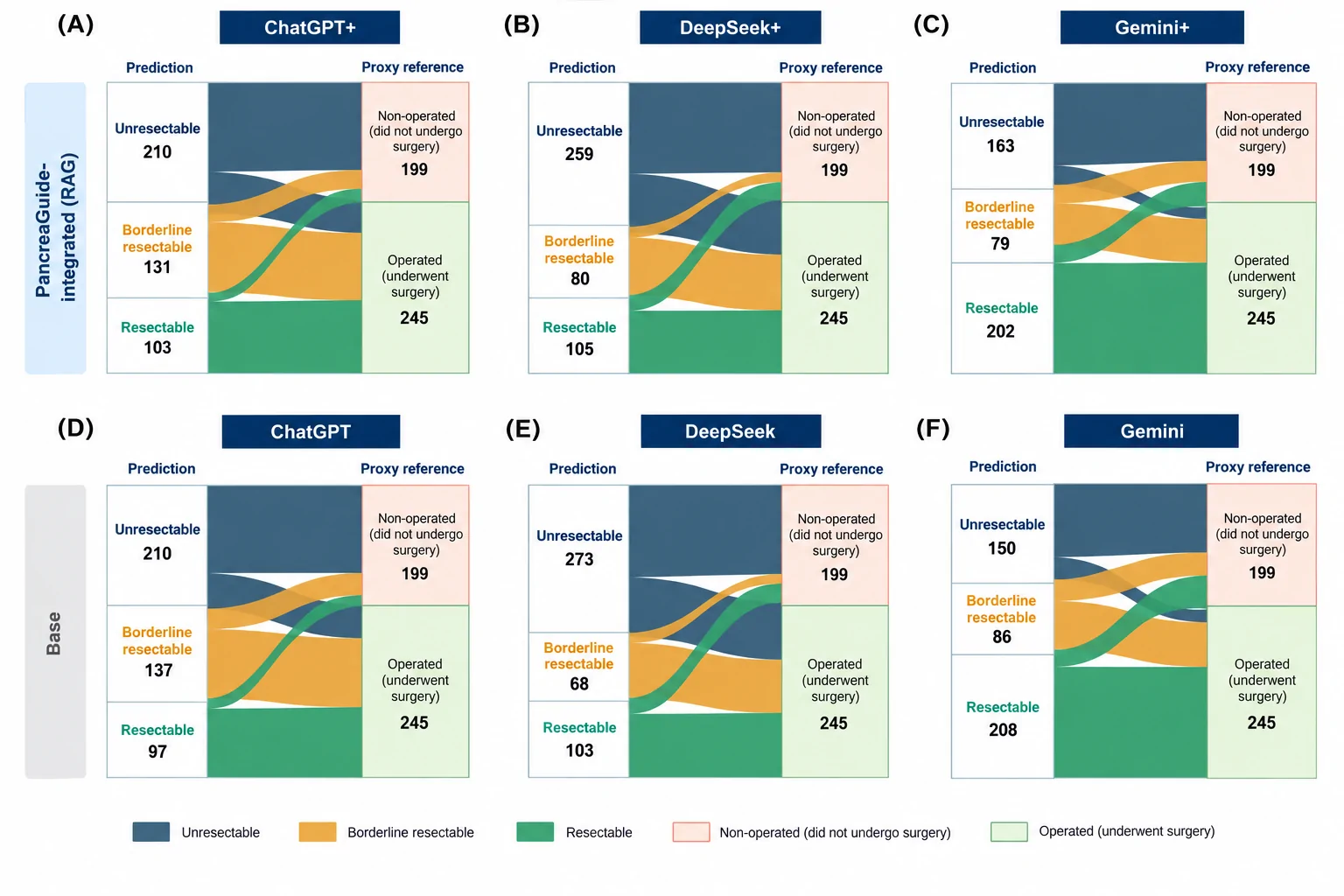
Agreement between model-generated resectability-related interpretations and observed surgical management status. Sankey diagrams illustrate the flow from model-predicted categories (unresectable, borderline resectable, and resectable) to observed surgical management status (nonoperated and operated). The upper row shows PancreaGuide-integrated models, and the lower row shows base models. (A-F) Columns represent different models (ChatGPT, DeepSeek, and Gemini). The width of each flow is proportional to the number of patients in each category. Ribbon widths represent the number of model-case predictions (n=444 per model configuration).

ROC curves are presented in Supplementary Figure 2B in Multimedia Appendix 1. PancreaGuide-integrated models showed higher discrimination estimates for agreement with the observed surgical management status, with curves generally positioned closer to the upper-left corner compared with base models. DCA (Supplementary Figure 2A in Multimedia Appendix 1) suggested a higher estimated decision-analytic net benefit across a range of threshold probabilities under the surgery-based proxy reference. However, because the reference standard was observed surgical status rather than independently adjudicated NCCN-defined resectability, these findings should not be interpreted as evidence that PancreaGuide directly improves surgical decision-making or formal resectability assessment. Subgroup analysis showed that PancreaGuide-integrated models had higher accuracy point estimates in both nonoperated and operated cohorts (Supplementary Figure 2C in Multimedia Appendix 1). In the operated group, Gemini+ achieved the highest accuracy (95.9%, 95% CI 0.927‐0.978), indicating higher sensitivity in classifying patients who ultimately underwent surgery. In the nonoperated group, ChatGPT+ and DeepSeek+ demonstrated more balanced performance, reflecting higher specificity in classifying patients who were managed nonoperatively.

### Error-Source Analysis

A structured case-level error-source analysis was performed for FP and FN classifications generated by PancreaGuide-integrated models. Because the same patient could be misclassified by more than one model, the unit of analysis was defined as the model–case error event. A total of 233 error events were independently reviewed by 2 senior pancreatic surgeons and assigned to predefined error-source categories. After harmonization of 2 synonymous category labels, the reviewers agreed on 153 of 233 (65.7%) events, with a Cohen κ value of 0.580, indicating moderate interrater agreement. Agreement was higher for FP events than for FN events (71/99, 71.7% vs 82/134, 61.2%), as shown in Supplementary Figure 3 in Multimedia Appendix 1. The remaining discordant events were adjudicated by a third reviewer, and final error-source categories were used to generate the final error distribution. The final distribution of error sources is summarized in Table S2 in Multimedia Appendix 1.

### Overall Performance of the Perioperative Consultation Module

The performance of LLMs in perioperative patient consultation was evaluated across 5 dimensions, including accuracy, completeness, consistency, safety, and robustness. As shown in Figure 6A, PancreaGuide-integrated models received higher expert-rated scores than their corresponding base models across all evaluated dimensions. The overall response quality is summarized in Figure 6B. Among all models, Gemini+ achieved the highest mean score (3.88, SD 0.38), followed by ChatGPT+ (mean 3.81, SD 0.36) and DeepSeek+ (mean 3.75, SD 0.35). In contrast, base models showed substantially lower performance, with DeepSeek exhibiting the lowest mean score (2.31, SD 0.58). Detailed dimensional trends are presented in Figure 6C, where PancreaGuide-integrated models demonstrated consistently higher scores across all 5 evaluation dimensions. Among these, improvements were most evident in completeness and robustness. The distribution of overall scores is illustrated in Figure 6D, showing a clear shift toward higher score ranges for integrated models, indicating improved response quality and consistency. Interrater reliability of expert evaluations was good across all 5 evaluation dimensions. The mean-rating ICC(2,3) values ranged from 0.801 to 0.880, with an overall ICC(2,3) of 0.850 (95% CI 0.840‐0.860), indicating good agreement among the 3 reviewers, as shown in Table S3 in Multimedia Appendix 1.

**Figure 6.**
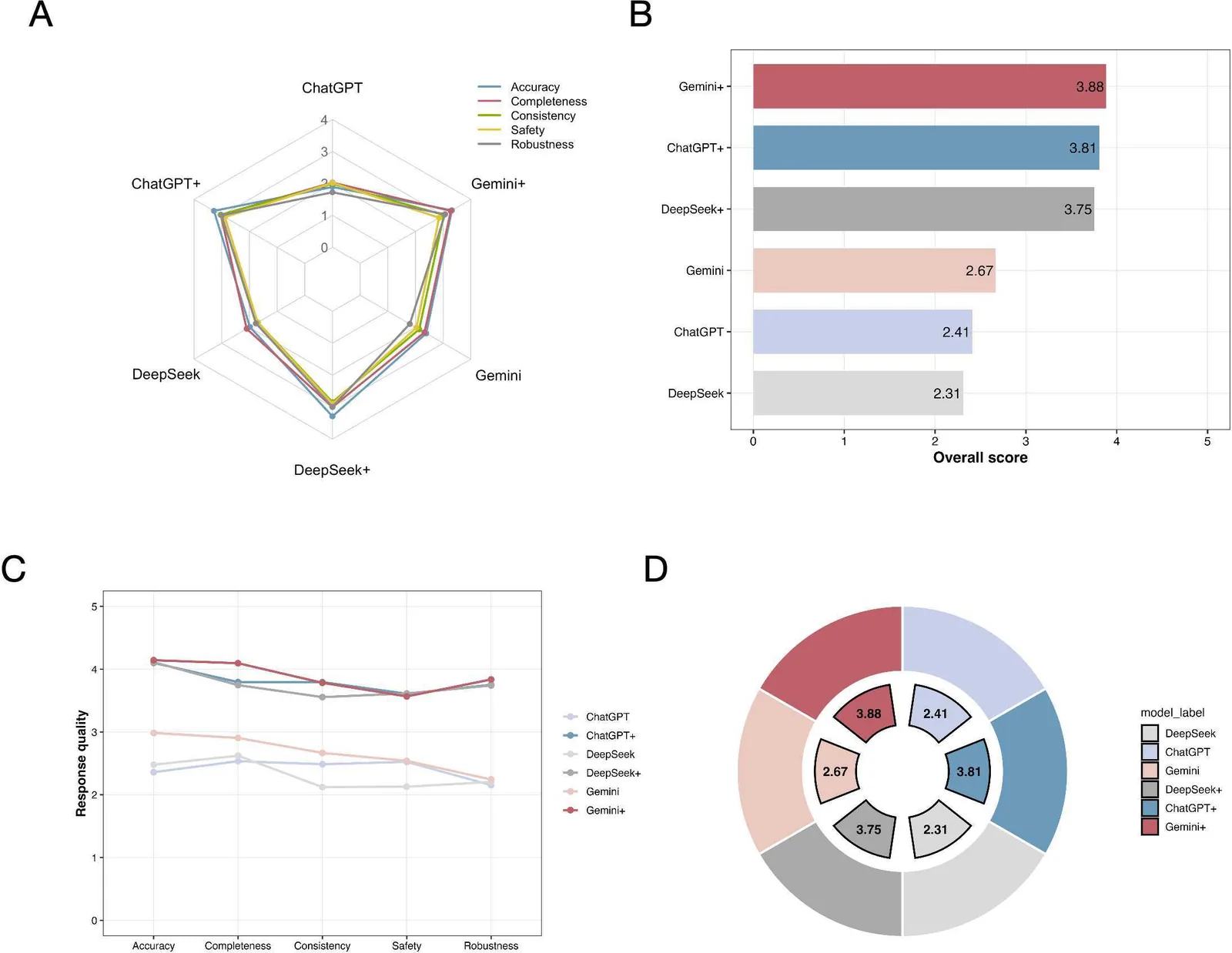
Evaluation of perioperative consultation performance across models. (A) Radar chart comparing model performance across 5 dimensions: accuracy, completeness, consistency, safety, and robustness. (B) Horizontal bar chart showing mean overall scores for each model. (C) Line plot illustrating performance across the evaluated dimensions. (D) Sunburst chart visualizing the distribution of overall scores among models. Models with the “+” suffix denote PancreaGuide-integrated versions.

### Domain-Specific Performance

The performance of models across different perioperative clinical domains is presented in Figure 7A. PancreaGuide-integrated models achieved higher mean scores across all evaluated categories, including surgical care, postoperative recovery, complication management, diet and nutrition, and prognosis and follow-up. Score distributions across these domains are shown in Figure 7B, where integrated models demonstrated higher median values than their corresponding base models. The differences between base and PancreaGuide-integrated models are summarized in Figure 7C, with all models showing consistent gains across categories. The largest improvements were observed in postoperative recovery and diet and nutrition domains. Finally, Figure 7D illustrates cross-domain consistency, showing that PancreaGuide-integrated models maintained stable performance across all categories, whereas base models exhibited greater variability.

**Figure 7.**
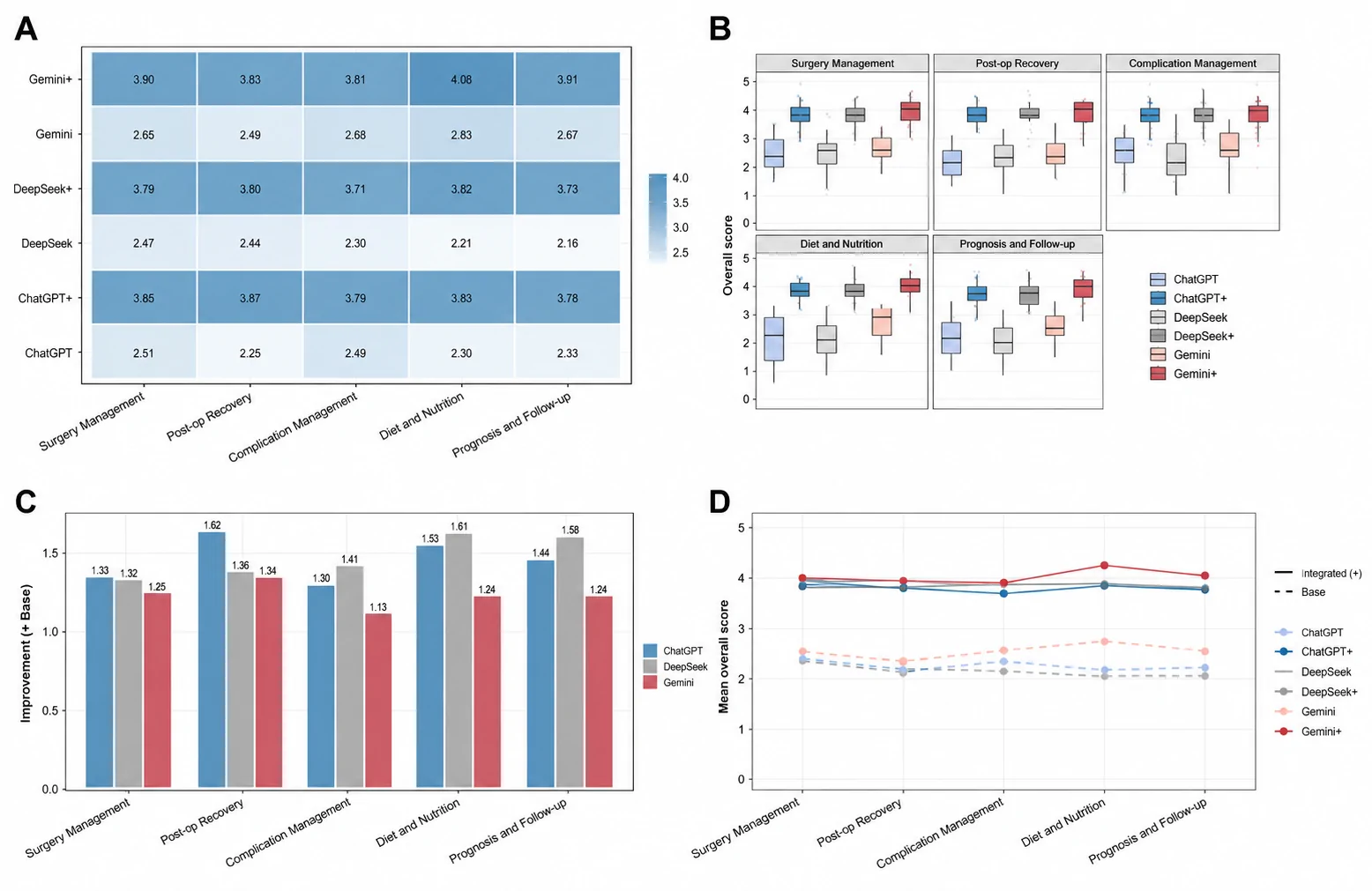
Domain-specific evaluation of perioperative consultation performance across models. (A) Heatmap showing mean scores across 5 clinical domains, including surgery management, postoperative recovery, complication management, diet and nutrition, and prognosis and follow-up. (B) Boxplots illustrating the distribution of scores across domains for each model. (C) Bar plots showing performance improvement of integrated models compared with base models across domains. (D) Line plots showing mean overall scores across domains. Models with the “+” suffix denote PancreaGuide-integrated versions.

## Discussion

### Principal Findings

In this retrospective study, we developed and evaluated PancreaGuide, a knowledge-augmented LLM framework designed for 2 clinician-supervised applications in pancreatic cancer care: the interpretation of resectability-related information from unstructured CT reports and standardized perioperative consultation support. Across 3 LLM backbones, PancreaGuide-integrated models showed improved agreement with observed surgical management status under the surgery-based proxy reference and received higher expert-rated scores for perioperative consultation. These findings support the feasibility of applying a unified LLM framework to both report-based information interpretation and patient-oriented perioperative education. However, the CT-based results reflect agreement with observed surgical management status rather than validation against independently adjudicated anatomical resectability.

Most previous medical LLM studies have focused on isolated tasks, such as question answering, report summarization, or structured information extraction [23,24]. PancreaGuide differs by integrating CT report interpretation and perioperative consultation into a common framework. This design reflects the practical continuity of pancreatic cancer care, in which patients and clinicians must interpret complex imaging descriptions while also addressing perioperative questions regarding treatment preparation, complications, nutrition, recovery, and follow-up. The present findings suggest that an integrated framework can generate more consistently structured outputs across these related information-support tasks. Nevertheless, the study did not directly evaluate whether this integration improved patient understanding, communication efficiency, shared decision-making, or clinical outcomes.

From a methodological perspective, PancreaGuide combines structured prompting, retrieval-augmented generation, task routing, reranking, and a domain-specific pancreatic surgery knowledge base. Retrieval-augmented generation may help constrain model responses to relevant medical information and has been increasingly used in medical LLM applications [25-27]. In the present study, higher performance estimates were observed after integration of the complete PancreaGuide framework. However, component-level ablation was not performed. Therefore, the independent contributions of structured prompting, retrieval, routing, reranking, postprocessing, and the knowledge base cannot be determined, and the observed differences should not be attributed to any single component [8,28,29].

A practical feature of PancreaGuide is that it operates on routinely available radiology reports rather than requiring direct access to raw CT images. Existing radiomics and deep learning approaches for pancreatic cancer assessment commonly rely on image preprocessing, tumor segmentation, and specialized computational infrastructure [30,31]. However, the model can only interpret information documented in the report. It cannot independently verify radiological findings, identify unreported abnormalities, or reassess tumor-vascular relationships from the original CT images [32]. PancreaGuide should therefore be regarded as an information interpretation tool rather than a substitute for specialist radiological review.

Under the surgery-based proxy reference, PancreaGuide-integrated models showed higher sensitivity and specificity point estimates than their corresponding base models. Higher sensitivity reflected greater agreement with patients who ultimately underwent surgery, whereas higher specificity reflected greater agreement with patients managed nonoperatively. These findings require cautious interpretation because surgical management status is influenced not only by anatomical resectability but also by patient preference, comorbidities, performance status, neoadjuvant treatment strategy, disease progression, institutional practice, and clinician judgment [33]. Accordingly, disagreement between model output and observed management may represent model error, incomplete report information, or discordance between imaging-defined resectability and the eventual management pathway. The present results do not establish that PancreaGuide can independently identify surgical candidates or improve treatment selection.

The confusion matrices showed fewer FP and FN classifications for PancreaGuide-integrated models under the proxy reference. However, classification counts alone do not explain the underlying causes of disagreement [34]. The structured error-source analysis demonstrated that misclassifications arose from multiple mechanisms rather than a single failure pattern. Ambiguous vascular involvement was the most frequent source of both FN and FP events, accounting for 31.3% of errors in each category. Treatment-related changes after neoadjuvant therapy represented an important contributor to FP classifications, whereas incomplete or indeterminate metastatic reporting, borderline terminology, and model extraction or interpretation errors also contributed to disagreement. Importantly, discordance introduced by the surgery-based proxy reference accounted for 12.1% of FP events and 15.7% of FN events. These findings highlight that disagreement with observed surgical management status does not necessarily indicate an incorrect interpretation of anatomical resectability by the model but may also reflect limitations of the proxy reference or incomplete clinical information [35]. Systematic case-level analysis is therefore needed to distinguish report-related ambiguity, model extraction errors, interpretation errors, and proxy-reference discordance, as these error types may have different clinical implications.

The perioperative consultation module also received higher expert-rated scores across accuracy, completeness, consistency, safety, and robustness. The largest score increases were observed in completeness and robustness, particularly for postoperative recovery and diet and nutrition. These findings indicate that the integrated models generated responses that were rated more favorably by pancreatic surgeons under the predefined scoring system. However, expert-rated response quality does not necessarily translate into improved patient comprehension, satisfaction, adherence, reduced anxiety, or the ability to distinguish general educational information from individualized medical advice. Prospective patient-level evaluation remains necessary.

Several limitations should be acknowledged. First, this was a retrospective, single-center study conducted at a high-volume tertiary pancreatic surgery center, and all CT reports were generated within a single institutional reporting system and in Chinese, which may limit generalizability across different languages, institutions, and reporting practices. Second, the observed surgical management status was used as a surgery-based proxy reference rather than an independently adjudicated, NCCN-defined anatomical resectability reference. Surgical management may be influenced by multiple nonanatomical factors, including patient preference, comorbidities, performance status, neoadjuvant treatment strategies, disease progression, institutional practices, and clinician judgment; therefore, the findings should be interpreted as agreement with observed management status rather than direct validation of anatomical resectability. Third, PancreaGuide was developed to interpret information documented in CT reports rather than raw imaging data or complete multidisciplinary clinical information, and the attribution of some error sources may remain uncertain without independent image rereview and comprehensive clinical adjudication. Fourth, because PancreaGuide was evaluated as an integrated framework, component-level ablation was not performed, and the independent contributions of structured prompting, retrieval augmentation, routing, reranking, postprocessing, and the knowledge base cannot be separately determined. Finally, the perioperative consultation module was assessed using expert-rated scoring rather than direct patient-level evaluation, and potential overlap between evaluation questions and knowledge-base content could not be completely excluded. Future multicenter studies incorporating independent resectability adjudication, multimodal imaging analysis, component-level validation, and prospective patient-centered evaluation are warranted.

### Conclusions

PancreaGuide provides a unified framework for clinician-supervised CT report interpretation and perioperative educational support in pancreatic cancer. Integration of the complete framework was associated with higher agreement with observed surgical management status and higher expert-rated consultation scores across multiple LLM backbones. These findings support further evaluation of PancreaGuide as an auxiliary information-support system but do not establish diagnostic validity for anatomical resectability or direct clinical benefit.

## Supplementary material

10.2196/101607 Multimedia Appendix 1 PancreaGuide development, implementation, and evaluation.

## Abbreviations

AUC: area under the curve
CT: computed tomography
DCA: decision curve analysis
FN: false negative
FP: false positive
ICC: intraclass correlation coefficient
LLM: large language model
NCCN: National Comprehensive Cancer Network
NPV: negative predictive value
PPV: positive predictive value
PSKB: pancreatic surgery knowledge base
Q&A: question-answering
RAG: retrieval-augmented generation
ROC: receiver operating characteristic
TN: true negative
TP: true positive
