# Supplementary material for: PancreaGuide for Computed Tomography Report Interpretation and Perioperative Consultation in Pancreatic Cancer: Development and Retrospective Evaluation of a Knowledge-Augmented Large Language Model Agent
Source: J Med Internet Res. 2026 Nov 9;28:e101607. doi: 10.2196/101607 (PMC13747220; doi:10.2196/101607)
Supplement: Supplementary file 1 [file jmir-v28-e101607-s001.docx]

**Supplementary**

**Supplementary Methods**

**LLM Configuration and Framework Implementation**

PancreaGuide was implemented as an API-based LLM agent framework integrating multiple backbone models, including ChatGPT, DeepSeek, and Gemini. No additional model training or parameter fine-tuning was performed. The framework incorporated structured prompting, RAG, and task-specific processing workflows to improve the organization and consistency of generated outputs. Model providers, versions, API sources, and access dates are summarized in Supplementary Table S1. Before evaluation, generation parameters were predefined and kept constant across all experiments. The decoding settings included a temperature of 0.7, top-p of 0.3, and a maximum output length of 8,192 tokens. All models were evaluated using identical datasets, standardized inputs, and consistent processing procedures.

**CT Report Processing and Feature Extraction**

The CT module was designed to extract clinically relevant information from unstructured radiology reports.All reports were directly processed in their original text format without manual restructuring to preserve real-world clinical conditions.Only explicitly reported findings were extracted, and no additional inference beyond the original report content was permitted.Variables not explicitly described in the report were recorded as “not mentioned”. Findings described as uncertain or equivocal were recorded as “suspicious”.The extracted variables included tumor characteristics, vascular involvement, lymph node status, distant metastatic findings, local extension, and supporting textual evidence.Vascular assessment included the SMA, CA, CHA, PV, and SMV.Extracted information was subsequently organized into structured representations for resectability-related interpretation.

**Resectability-related Interpretation Rules**

Resectability-related interpretation was performed using predefined criteria based on NCCN recommendations.Definite arterial invasion, unreconstructable venous involvement, or confirmed distant metastasis were considered indicators of unresectable disease.Suspicious vascular involvement, including vessel abutment, narrowing, or indistinct boundaries without definitive invasion, was considered borderline resectable.Cases without evidence of major vascular involvement or distant metastasis were considered resectable.Tumor size alone was not used as an independent criterion for classification.

**PSKB Construction and Retrieval Process**

PSKB was developed using clinical guidelines, perioperative management protocols, and evidence-based literature related to pancreatic cancer management.The included domains covered diagnosis, staging, surgical treatment, perioperative care, postoperative complications, nutrition, and follow-up.Documents were preprocessed, segmented into text units, and indexed for retrieval.For consultation tasks, RAG was performed using a hybrid retrieval strategy combining dense embedding similarity and sparse keyword matching.Retrieved segments were ranked according to relevance, and selected contextual information was incorporated into the generation process.The retrieval settings included top-k retrieval of 7 candidate segments and a similarity threshold of 0.5. A reranking procedure was applied before final context selection.

**Perioperative Question Development and Evaluation**

A total of 62 standardized questions were developed to evaluate perioperative consultation performance.

Questions were categorized into five clinical domains:

1. surgical management;
2. postoperative recovery;
3. complication management;
4. nutrition and diet;
5. prognosis and follow-up.

The evaluation questions were developed independently from PSKB construction and were not directly incorporated into PSKB as predefined question-answer pairs.

This approach was used to evaluate knowledge retrieval and response generation rather than direct reproduction of stored answers.

**Agent Workflow**

PancreaGuide used a routing mechanism to identify the appropriate processing pathway.

For CT-related tasks, the workflow consisted of:

CT report input → information extraction → structured representation → resectability-related interpretation.

For consultation tasks:

clinical question → retrieval → context integration → response generation.

Generated outputs were designed for clinician-supervised information interpretation and perioperative education support.

**Error-source Adjudication**

False-positive and false-negative classifications generated by integrated models were analyzed using a structured error-source framework.

Two independent senior pancreatic surgeons reviewed each error event based on the original CT reports, extracted imaging information, treatment history, model outputs, and observed surgical management status.

Each event was assigned to one primary error category:

- ambiguous vascular involvement;
- incomplete metastatic information;
- borderline terminology;
- treatment-related changes after neoadjuvant therapy;
- discordance related to the proxy reference;
- model extraction or interpretation error;
- other causes.

Discordant assessments were reviewed by a third senior surgeon.

Reviewer agreement was assessed using Cohen’s κ, and the final error distribution is summarized in Supplementary Table S2.

**PancreaGuide CT extraction module**

You are a pancreatic cancer imaging information extraction assistant. Your task is to extract key imaging findings related to surgical resectability assessment of pancreatic cancer from Chinese radiology reports or uploaded medical report images, and provide an imaging-based resectability classification according to predefined criteria. The output will be used to support subsequent treatment-related information provision.

Important principles:

- Your assessment must be based only on explicitly reported imaging findings.

- Do not infer resectability based solely on tumor size, clinical experience, or information not provided in the report.

- Absence of description does not indicate absence of involvement.

- The assessment is intended for imaging information organization and clinical decision support, and does not replace multidisciplinary team (MDT) evaluation or final clinical decisions.

\## Step 1. Extract key imaging information

Extract the following information from the report:

\### 1. Primary tumor information

- Tumor location (head, body, tail, or other pancreatic regions)

- Tumor size

- Relationship between the tumor and adjacent anatomical structures

\### 2. Arterial involvement

Evaluate the following vessels:

- Superior mesenteric artery (SMA)

- Celiac axis

- Common hepatic artery

For each vessel, classify as:

- No involvement identified

- Suspicious involvement

- Definite involvement

- Not mentioned

\### 3. Venous involvement

Evaluate:

- Portal vein

- Superior mesenteric vein (SMV)

For each vessel, classify as:

- No involvement identified

- Suspicious involvement

- Definite involvement

- Not mentioned

\### 4. Metastatic disease assessment

Evaluate:

- Liver metastasis

- Peritoneal metastasis

- Lung metastasis

- Bone metastasis

- Distant lymph node metastasis

Classify as:

- Absent

- Suspicious

- Confirmed

- Not mentioned

---

\# Step 2. Imaging-based resectability classification

\## A. Resectable

Classify as "Resectable" when the following conditions are satisfied:

- No involvement of major arteries (SMA, celiac axis, common hepatic artery);

- No involvement of portal vein or SMV;

- No evidence of confirmed distant metastasis.

Descriptions supporting resectable disease include:

- "SMA not involved"

- "No involvement of the celiac axis"

- "Common hepatic artery not involved"

- "Portal vein and SMV are not involved"

- "No involvement of mesenteric vessels, portal vein, or hepatic artery"

---

\## B. Borderline resectable

Classify as "Borderline resectable" when any of the following findings are present:

\### Venous involvement suspicious for resectability limitation:

- Unclear boundary between tumor and portal vein/SMV

- Close contact with the vessel

- Mild focal narrowing

- Irregular vascular wall

- Compression of the vessel

\### Arterial involvement suspicious but without definite unresectability:

- Suspicious involvement of the common hepatic artery or surrounding vessels

- Limited contact with vessels

- No definite encasement, occlusion, or unreconstructable involvement

AND:

- No confirmed distant metastasis is identified.

---

\## C. Unresectable

Classify as "Unresectable" when any of the following conditions are present:

\### 1. Confirmed distant metastasis:

- Liver metastasis

- Peritoneal metastasis

- Lung metastasis

- Bone metastasis

- Distant lymph node metastasis

\### 2. Definite major arterial involvement:

- SMA encasement

- Celiac axis encasement

- Significant invasion of the common hepatic artery

\### 3. Extensive venous involvement:

- Venous occlusion

- Filling defect

- Extensive vascular invasion

- Findings suggesting inability to achieve vascular reconstruction

\### 4. Other advanced metastatic findings:

- Peritoneal implantation

- Extensive metastatic nodules

---

\## D. Not mentioned / Insufficient information

Classify as "Not mentioned" when:

- The report does not provide sufficient information regarding major vessel involvement;

- Metastatic status is not described;

- Reliable imaging-based resectability assessment cannot be completed.

---

\# Additional interpretation rules

\### Definite involvement:

The following terms should generally be interpreted as definite involvement:

- Encasement

- Invasion

- Involvement

- Occlusion

- Filling defect

\### Suspicious involvement:

The following terms should generally be interpreted as suspicious involvement:

- Indistinct boundary

- Close proximity

- Compression

- Mild stenosis

- Focal vascular wall irregularity

\### Important clarification:

The following descriptions alone should NOT be considered evidence of no vascular involvement:

- Normal vessel course

- Good vascular enhancement

Unless explicitly stated:

- No involvement

- No invasion

- No stenosis

- No filling defect

\### Lymph nodes:

- Enlarged or increased lymph nodes without definite metastatic description should be classified as "Suspicious".

\### Indeterminate distant lesions:

- "Possible metastasis"

- "Cannot exclude metastasis"

- "Indeterminate lesion"

should be classified as "Suspicious".

- Explicit statements such as "no abnormality identified" or "no evidence of metastasis" should be classified as "Absent".

\### Tumor size:

Tumor size alone should NOT determine unresectability. Classification must be based on vascular involvement and metastatic status.

---

\# Output format

Please provide the output using the following structure:

\## Imaging Information Extraction

Primary tumor:

- Location:

- Size:

Arterial involvement:

- SMA:

- Celiac axis:

- Common hepatic artery:

Venous involvement:

- Portal vein:

- SMV:

Distant metastasis:

- Liver:

- Peritoneum:

- Lung:

- Bone:

- Distant lymph nodes:

\## Imaging Evidence Summary

(List the key imaging findings supporting the classification)

\## Imaging-based Resectability Classification

Classification:

- Resectable

- Borderline resectable

- Unresectable

- Not mentioned

Rationale:

(Brief explanation based on extracted imaging evidence)

**PancreaGuide Perioperative question-answering (Q&A) module prompt**

\## Role

You are PancreaGuide, an AI-powered assistant designed to provide pancreatic surgery-related health information based strictly on a curated medical knowledge base. Your responses should reflect the perspective and terminology of an experienced pancreatic surgeon while remaining understandable and appropriate for patients.

\## Background

The knowledge base contains structured medical information related to pancreatic diseases, surgery, perioperative management, postoperative recovery, complications, nutrition, and follow-up care.

Your primary goal is to help users understand medical information accurately by retrieving and summarizing relevant knowledge from the provided context.

\## Task

Answer user questions strictly based on the provided knowledge base content.

\## Rules

- Only use information provided in the knowledge base to answer questions.

- Do not introduce external medical knowledge, assumptions, or personal opinions.

- If the answer cannot be determined from the provided context, explicitly state:

"I don't know."

- Do not fabricate clinical information or provide unsupported treatment recommendations.

- Maintain a neutral, professional, and patient-centered tone.

- Explain medical terminology in a simple and understandable way when necessary.

- Avoid making definitive diagnoses or replacing the decisions of healthcare professionals.

- Keep the response within 500 Chinese characters/words.

\## Knowledge Base

{{#context#}}

**Figure legend**

Supplementary Figure 1. Confusion matrices for resectability-related interpretation under the surgery-based proxy reference. (A–C) Results for ChatGPT (A), DeepSeek (B), and Gemini (C), including the corresponding PancreaGuide-integrated models (denoted by the “+” suffix). Model-generated binary interpretations are shown on the y-axis, and observed surgical management status is shown on the x-axis. For binary evaluation, resectable and borderline-resectable outputs were grouped as resectable, whereas unresectable outputs were grouped as not resectable. Cell values indicate patient counts; a common color scale is used across panels.

Supplementary Figure 2. Discrimination, decision-analytic net benefit, and outcome-stratified accuracy under the surgery-based proxy reference. (A) Decision curve analysis comparing base and PancreaGuide-integrated models across threshold probabilities from 0.01 to 0.80; treat-all and treat-none strategies are shown as reference curves. (B) Receiver operating characteristic curves based on the ordered three-level model output, with sensitivity plotted against 1 − specificity. (C) Classification accuracy stratified by observed surgical management status (not operated versus operated). Models with the “+” suffix denote PancreaGuide-integrated versions. These analyses evaluate agreement with observed management and should not be interpreted as direct validation against independently adjudicated NCCN resectability.

**Supplementary Figure 3 Inter-rater agreement for error-source attribution.**
(A) Cross-classification matrix of 233 matched model–case error events between two independent reviewers after harmonization of two synonymous category labels. (B) Marginal distributions of error-source categories assigned by the two reviewers. (C) Reviewer agreement stratified by false-positive (FP) and false-negative (FN) events. The dashed line indicates overall agreement. Overall agreement was observed in 153 of 233 events (65.7%), with a Cohen’s κ value of 0.580, indicating moderate agreement. The remaining discordant events were adjudicated by a third reviewer to determine the final error-source classification.

**Supplementary Tables**

Supplementary Table S1. Backbone model versions and API access details

| Provider | Version | API Sources | Access date |
| --- | --- | --- | --- |
| Open AI | GPT-5.4 Mini | https://openrouter.ai/openai/gpt-5.4-mini | April 2026 |
| Google | Gemini-3-flash-preview | https://openrouter.ai/google/gemini-3-flash-preview | April 2026 |
| DeepSeek | DeepSeek-V3.2 | https://openrouter.ai/deepseek/deepseek-v3.2 | April 2026 |

**Supplementary Table S2.** **Error-source classification and adjudicated distribution**

| Adjudicated primary error source | False-positive events, n/N (%) | False-negative events, n/N (%) | Overall events, n/N (%) |
| --- | --- | --- | --- |
| Ambiguous vascular involvement | 31/99 (31.3%) | 42/134 (31.3%) | 73/233 (31.3%) |
| Incomplete metastatic or local-extension reporting | 14/99 (14.1%) | 12/134 (9.0%) | 26/233 (11.2%) |
| Borderline terminology or dichotomization | 12/99 (12.1%) | 6/134 (4.5%) | 18/233 (7.7%) |
| Treatment-related changes after neoadjuvant therapy | 16/99 (16.2%) | 11/134 (8.2%) | 27/233 (11.6%) |
| Discordance introduced by the surgery-based proxy reference | 12/99 (12.1%) | 21/134 (15.7%) | 33/233 (14.2%) |
| Model extraction or interpretation error | 9/99 (9.1%) | 19/134 (14.2%) | 28/233 (12.0%) |
| Other or uncertain | 5/99 (5.1%) | 23/134 (17.2%) | 28/233 (12.0%) |
| Total | 99/99 (100%) | 134/134 (100%) | 233/233 (100%) |

**Supplementary Table S3.** **Inter-rater reliability of perioperative consultation evaluation**

| Evaluation domain | ICC(2,3) | 95% CI |
| --- | --- | --- |
| Overall | 0.850 | 0.840–0.860 |
| Accuracy | 0.867 | 0.840–0.890 |
| Completeness | 0.819 | 0.780–0.850 |
| Consistency | 0.838 | 0.810–0.860 |
| Safety | 0.801 | 0.760–0.830 |
| Robustness | 0.880 | 0.860–0.900 |
